# Improving Phrap-Based Assembly of the Rat Using “Reliable” Overlaps

**DOI:** 10.1371/journal.pone.0001836

**Published:** 2008-03-19

**Authors:** Michael Roberts, Aleksey V. Zimin, Wayne Hayes, Brian R. Hunt, Cevat Ustun, James R. White, Paul Havlak, James Yorke

**Affiliations:** 1 Institute for Physical Science and Technology, University of Maryland, College Park, Maryland, United States of America; 2 Human Genome Sequencing Center, Baylor College of Medicine, Houston, Texas, United States of America; University of Liverpool, United Kingdom

## Abstract

The assembly methods used for whole-genome shotgun (WGS) data have a major impact on the quality of resulting draft genomes. We present a novel algorithm to generate a set of “reliable” overlaps based on identifying repeat k-mers. To demonstrate the benefits of using reliable overlaps, we have created a version of the Phrap assembly program that uses only overlaps from a specific list. We call this version *PhrapUMD*. Integrating PhrapUMD and our “reliable-overlap” algorithm with the Baylor College of Medicine assembler, Atlas, we assemble the BACs from the *Rattus norvegicus* genome project. Starting with the same data as the Nov. 2002 Atlas assembly, we compare our results and the Atlas assembly to the 4.3 Mb of rat sequence in the 21 BACs that have been finished. Our version of the draft assembly of the 21 BACs increases the coverage of finished sequence from 93.4% to 96.3%, while simultaneously reducing the base error rate from 4.5 to 1.1 errors per 10,000 bases. There are a number of ways of assessing the relative merits of assemblies when the finished sequence is available. If one views the overall quality of an assembly as proportional to the inverse of the product of the error rate and sequence missed, then the assembly presented here is seven times better. The UMD Overlapper with options for reliable overlaps is available from the authors at http://www.genome.umd.edu. We also provide the changes to the Phrap source code enabling it to use only the reliable overlaps.

## Introduction

Most genomes for which draft assemblies are available have been assembled using the whole-genome shotgun (WGS) method or a hybrid-WGS technique. In the WGS method many copies of the genome are randomly fractured into fragments, with estimated lengths that usually run from several thousand bases (Kb) (plasmids and fosmids) to some that are well over 100 Kb (Bacterial Articial Chromosomes or BACs). The actual length of each fragment is likely to differ from the estimated length by perhaps 10% to 20%. The sequences of the two ends of each fragment are then read imperfectly. The sequence of each end is called a *read*. Two reads that were created from opposite ends of the same fragment are said to be *mates*, and they form a *mate-pair*. Each read has up to 1000 bases. As the sequence is created, each base is assigned a quality score related to the probability that the base is being reported incorrectly [1], [2]. Enough fragments are created so that a typical base in the genome is represented in several reads, usually about seven to thirteen. Given this data and no more, the WGS assembly problem is to assemble the genome as completely and correctly as possible. Several genome assembly programs have been developed, such as the TIGR assembler [3], the Celera Assembler [4], Atlas [5], Arachne [6], Phusion [7], JAZZ [8], and PCAP [9]. Although appearing deceptively simple, genome assembly is remarkably difficult in practice. This is evident by the fact that despite using the same input, different assemblers can produce draft assemblies that differ considerably in size and error rates.

Several assembly programs (e.g. Phusion and Atlas) utilize Phrap [10] at the early stages of the assembly. Phrap is also widely used as a standalone tool for creating local assemblies of the BAC-sized (up to about 250K bases) regions of genomic sequence. Given a set of reads and optional quality scores, Phrap computes overlaps and assembles the reads into contigs, generating a read multi-alignment, a contig sequence and sequence quality information. We have produced *PhrapUMD*, a modified version of Phrap that allows the user to control which overlaps Phrap uses in building contigs. We paired PhrapUMD with the UMD Overlapper [11], which corrects errors in the reads and accurately computes a set of high-quality overlaps that we call “reliable”. This paper shows how a Phrap-based assembler can be improved by simply substituting the UMD Overlapper and PhrapUMD for Phrap in its pipeline. To demonstrate the power of our techniques, we integrated them into Atlas, the Baylor College of Medicine (Baylor) assembly program. We used the modified Atlas to produce assemblies of approximately 20,000 BACs from the rat genome project. We report here on how the modification improves Atlas' ability to assemble the genome of the rat *Rattus Norvegicus* [12].

We note that the methods that we propose and evaluate in this manuscript are mostly useful for assembly programs utilizing Phrap for building contigs. We do not expect that the use of “reliable” overlaps will bring about any improvements for assembly programs that do not use Phrap, such as Celera Assembler, Arachne, or PCAP. Still, there are many centers that use Phrap for assembling genomes or fragments of genomes, such as National Intramural Sequencing Center at NIH, Human Genome Sequencing Center at Baylor College of Medicine, Sanger Centre, and many others. The methods discussed in this paper will be of great benefit to these centers.

We evaluate our assembly methods by comparing the resulting draft with the finished sequence of a part of the genome. By commonly accepted definition, the finished sequence is a gapless sequence with less than 1 error per 10000 bases, whose validity has been checked and corrected with additional local sequencing. However, it is important to note that this sequence may not be completely correct [13].

## Methods

One of the first steps in creating an assembly from WGS data is to determine which reads overlap each other based on comparison of their sequences. The fact that two reads' sequences agree over some interval does not necessarily imply that these reads came from the same part of the genome. They might have come from different copies of a repetitive region. We call the set of all overlaps between reads *plausible*. Some portion of the plausible overlaps is spurious due to repetitive regions in the genomes. In this paper, we describe a technique that identifies a subset of the plausible overlaps that we call *reliable*. The reason for creating reliable overlaps is to avoid creating misassemblies at the early stages of the assembly when the contiguous chunks of sequence (contigs) are built using only overlap information.

**Figure 1:**
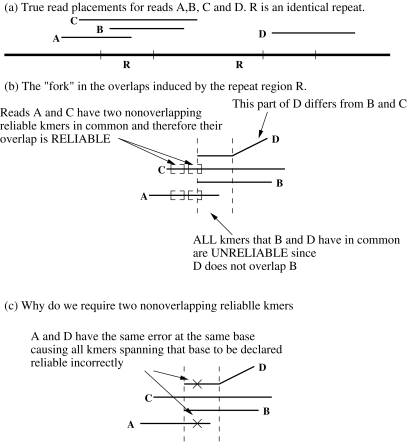
Illustration of the technique that identifies reliable overlaps: (a) a scenario where a genome contains two copies of a repeat region R. The correct positions of reads A, B, C and D are shown. (b) A “fork” in the overlaps. (c) a scenario where reads A and D have the same sequencing error at the same base.

Figure 1a shows a scenario where a genome contains two copies of a repeat region R. The correct positions of reads A, B, C and D are shown. The repeat region causes a “fork” in the overlaps, as shown in Figure 1b. The fork is created because read A has a plausible overlap with reads B, C and D, but D does not overlap B and C. We call the overlaps of A with B and D “fork overlaps”. Our goal is to design a method that eliminates the fork overlaps from the list of plausible overlaps, thereby producing a list of overlaps we call “reliable”. In Figure 1b, the only overlap that we would like to call reliable is between reads A and C, because part of the overlap region is outside the repeat region.

We accomplish the task of eliminating the fork overlaps by identifying the fork 20-mers. In Figure 1b all 20-mers belonging to the region between the dashed lines are considered to be “fork 20-mers” because they are present in reads B and D, which do not overlap. More generally, we define a 20-mer to be a “fork 20-mer” if there are two non-overlapping reads that have this 20-mer in common. We define a 20-mer to be “reliable” if it is not a fork 20-mer, that is if all reads containing the 20-mer plausibly overlap.

We define an overlap to be reliable if the reads have at least two non-overlapping reliable 20-mers in common (see Figure 1b). We might like to call an overlap reliable if the overlapping sequence contains even one reliable 20-mer, but this might be an illusion caused by sequencing errors. Figure 1c shows a scenario where reads A and D have the same sequencing error at the same base. For example, a C was read as a G in both reads at the same location (marked by a cross). This error will cause each 20-mer spanning the error's location to be declared reliable, assuming that only A and D contain these error-induced 20-mers, because A and D plausibly overlap. We impose the requirement of two non-overlapping reliable 20-mers to make sure that the overlap between A and D is not declared reliable because of a sequencing error. Our method will not declare the overlap between A and D to be reliable unless these reads have two sequencing errors of the same kind at two matching bases. In practice there are rare occasions in which a spurious overlap is labeled reliable, but as assembly results show, such overlaps do not cause major problems.

To test the effectiveness of using the UMD reliable overlaps with PhrapUMD, we incorporated our methods into Atlas, the Baylor College of Medicine assembly program. We applied the resulting software to the assembly of the rat genome. Atlas utilized the hybrid WGS – BAC approach to sequence the rat genome. Most of the rat genome was covered by a tiling of about 20,000 BACs, each averaging over 200 Kb of sequence. These BACs were individually sequenced at low coverage (generally 1x to 2x). The Atlas strategy was to consider the set of reads from each BAC (BAC reads), find which WGS reads appeared to overlap the BAC reads, and then add in these WGS reads and their mates. This approach resulted in independent data sets (buckets) such that an assembly could be created for each BAC. With these sets of reads, Atlas ran Phrap on each bucket to build contigs and then arranged the contigs into scaffolds using mate pair information. We assembled each BAC, but did not merge the scaffolds of the different BACs.

The UMD+Atlas results reported in the following section were obtained by incorporating the following UMD techniques into Atlas:

1. We use the UMD Overlapper [11] to determine plausible overlaps. Since the UMD Overlapper is capable of error correcting the reads, we trim reads only when the expected error rate reaches 10%, based of the reported quality scores. This process yields reads that are about 12% longer. We chose such trimming because it provided the longest contigs.
2. We determine reliable overlaps, and then use PhrapUMD to create a set of high quality contigs that we call *reliable* contigs. These are generally shorter than regular Phrap contigs, but they are lengthened in the following step.
3. After scaffolding with Atlas, we examine each pair of adjacent contigs to see if their ends would overlap according to the set of plausible overlaps produced in (1), if at most one read were removed from each end. If this is the case, we then create an extended set of overlaps consisting of the reliable overlaps combined with plausible overlaps of the end reads from the adjacent contigs. We find that a second pass of PhrapUMD using this slightly extended set of reliable overlaps results in much bigger contigs without sacrificing the error rate of the resulting sequence or the fraction of finished sequence covered. These contigs are then scaffolded with the Atlas scaffolder to get the final result. In this way, we effectively force PhrapUMD to use mate pair information to build contigs. The ability to limit the overlaps PhrapUMD may consider turns it into a tool that can be used iteratively.

We note that our method's ability to resolve repeats is still limited by the size of the largest insert library that is available. Any repeat that is larger than the longest library available may cause misassemblies. Original Phrap does not use mate pair information in building contigs. It would be very beneficial to implement some direct way to have Phrap use mate pairing data, but this would require major changes to the code and may result in reduced useability and stability of the software. One of the reasons why Phrap is so widely used is that it is stable and easy to install and run software, and our goal was to gain maximum improvement while introducing minimal changes to the Phrap software. Reliable overlaps allow Phrap to build “unitigs” (for more information on unitigs see [4]). Unitigs are contigs that can be assembled in a unique way, and thus repeat and unique regions are assembled into separate unitigs. The subsequent step of scaffolding the unitigs and then expanding the set of overlaps allows Phrap to indirectly use mate pair information in building its final contigs.

## Results and Discussion

In this paper, we use the data set Freeze02, a complete collection of read data, and a corresponding Atlas assembly of the rat produced by the Rat Genome Sequencing Consortium. We restrict our report to the subset of reads covering 4.3 million bases of finished sequence in 21 BACs. At the time this work was performed this was the largest contiguous chunk of finished sequence that was available to us. The average read coverage of the 21 BACs is about 7. For all 33 million reads, the average coverage is about 7.3. While 4.3Mb is only a bit more than 0.1% of the rat genome, it does provide a substantial test bed. Later data sets such as Freeze03 and Freeze04 incorporate finished sequence, so they cannot be used to test the skill of the WGS assembly techniques.

Following the Atlas standard, we consider only those contigs output by UMD+Atlas that are 1 Kb or longer. We then match these contigs against finished sequence using BLASTZ software [14]). We score each match. Experience has shown that if a contig has more than one BLASTZ match to finished sequence, the longer match is not necessarily the more desirable one. Often, another match with a slightly shorter length but many fewer errors will be present, and better alignments can be found by defining a score *S* that severely penalizes errors. If *K* is the factor by which we penalize each base error, we define the score of an alignment to be
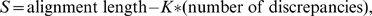
for each alignment. We use *K* = 125, which means that a successful match can have at most a 0.8% error rate, compared to finished sequence. The parameters we have used for Blastz comparisons are *C = 2 W = 16 T = 0 K = 25000*, where *K* relates to the the gap penalty (the default value for which is *K = 2500*, *W* is the word length used in initiating a match, and *C = 2* ensures that BLASTZ uses a “chain and extend" approach in matching sequences (the default is to not chain).We use all matches that are at least 1kb in length. If a contig matches in multiple places, we pick the match that has the highest positive score. The *tails* of a contig are the parts at either or both ends that are outside the successful match.

We measure the following quantities for each assembly:

- % Non-Matching Contig Tails: the percentage of assembly bases that are in non-matching tails of contigs. These reflect assembly errors on the ends of contigs.
- % of Finished Sequence Matched: the percentage of the span of finished sequence that is matched by contigs longer than 1 Kb. Erroneous bases are counted in this number. If a finished base is matched by more than one contig, the base is counted only once.
- Number of Contigs: total number of contigs in the scaffolds of the assembly of the 21 BACs.
- Interior Error rate: We take only the highest scoring alignment of a contig to the finished sequence as the match and define the interior error rate across a set of BACs to be
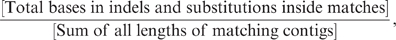
where the sums are carried out over all matching contigs in all BACs. (Note that the denominator here is the sum of the matching lengths of contigs rather than the number of finished bases covered, and that all errors in the contigs are counted. For example, if two contigs cover a given finished base, and both get the base wrong, then both errors are counted.) The cumulative results for a data set consisting of 21 BACs are presented in Table 1:
- The top line of the table gives the results of the original Atlas utilizing Phrap from the Freeze 02 assembly. The original Atlas assembly has an interior error rate of 0.045% and matches 93.4% of the finished sequence.
- The line, “original Atlas with UMD Plausible”, shows the result of substituting PhrapUMD with UMD *plausible* overlaps for Phrap in the original Atlas. The primary impact of the switch is that 2.7% more of the finished sequence is matched.
- The third line, “original Atlas with UMD Reliable”, shows the result of substituting PhrapUMD with UMD *reliable* overlaps for Phrap in the original Atlas. This assembly covers slightly more finished sequence, but more importantly, decreases both the interior error rate and the tail errors by roughly a factor of 4. However, the number of contigs increases to 480, i.e. the assembly becomes more fragmented.
- The forth line, “two-pass Atlas with UMD Reliable,” shows the result of using UMD reliable overlaps and the two-pass approach described in the methods section. This is our best assembly. At 1/4 the original Atlas error rate, this assembly has approximately 3% more bases matching finished sequence than the Atlas assembly.

**Table 1:** Comparison of the three assemblies for the subset of the 21 BACs from the Rat genome.

Assembly	% Non-Matching Contig Tails	% of Finished Sequence Matched	% Interior Error Rate	Number Of Conigs
original Atlas	0.331	93.4	0.045	377
original Atlas with UMD Plausible	0.448	96.1	0.041	375
original Atlas with UMD Reliable	0.118	96.3	0.012	480
two-pass Atlas with UMD Reliable	0.075	96.3	0.011	371

The “original Atlas with UMD Plausible” and “original Atlas with UMD reliable” assembly results obtained by substituting Phrap for PhrapUMD with UMD plausible and reliable overlaps respectively. The best assembly (the bottom line) uses PhrapUMD and UMD reliable overlaps utilizing the 2-pass approach described in the “Methods” section. It has almost 3% more sequence matching finished sequence than original Atlas with Phrap at less than 1/4 the original base error rate.

We note that the reduction in the interior error rate is mostly due to error-correction and trimming routines in the UMD Overlapper. By providing Phrap with trimmed and error-corrected reads, we reduce the possibility of errors in Phrap consensus.

**Figure 2:**
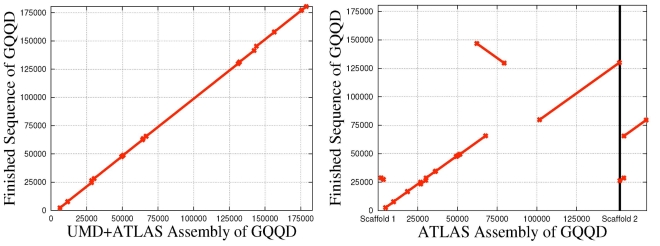
Two alignments of assemblies to the finished sequence of BAC GQQD. The original Atlas assembly created two scaffolds only covering 73.2% of the finished sequence. Note the misplaced 20 Kb segment in the Atlas assembly. The UMD+Atlas assembly of GQQD correctly places the 20 Kb section originally misplaced and creates a single scaffold of the BAC covering 93.3% of the finished sequence. This UMD+Atlas assembly used reliable overlaps. This was the BAC that gave Atlas the most trouble.

**Figure 3:**
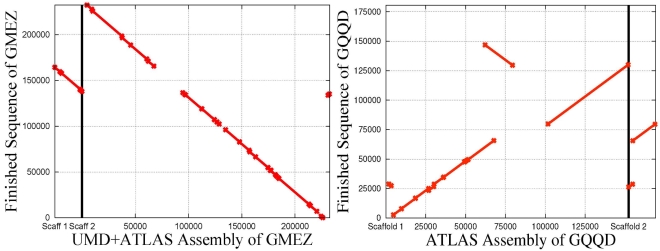
Two alignments of assemblies to the finished sequence of BAC GMEZ. The original Atlas assembly created a single scaffold. The UMD+Atlas assembly of GMEZ assembled a 26 Kb section from the middle of the bigger scaffold into a separate Scaffold 1. Note that the large scaffold gap in the Scaffold 2 is estimated correctly. This UMD+Atlas assembly used reliable overlaps. This was the BAC that gave UMD+Atlas the most trouble and the only case where UMD+Atas assembly had two scaffolds.

Figure 2 shows one of our most dramatically improved BACs. We used NUCmer, a variant of the MUMmer program [15], to align the assemblies of the BAC GQQD to the finished sequence. This particular BAC was initially assembled by Atlas into two scaffolds, and one scaffold contained a 20 Kb section that was reversed and misplaced. Using PhrapUMD with reliable overlaps, UMD+Atlas assembled the entire BAC into one scaffold and fixed the major misassembly. Our assembly of this BAC matched 20.0% more finished sequence than the Atlas assembly and reduced the interior error rate from 4.3 errors per 10 Kb in the Atlas assembly to 1.7 errors per 10 Kb. Figure 3 demonstrates the worst UMD+Atlas assembly. This was the only BAC that got assembled into two separate scaffolds; the rest of them were assembled into a single scaffold. In this BAC a 26Kb section in the middle was assembled into a separate scaffold, Scaffold 1, whereas the rest of the BAC was assembled into Scaffold 2. The gap in the middle of Scaffold 2, matching the size and position of the Scaffold 1, was estimated correctly. We do not view this scenario as a misassembly.
